# Chronic disease outcomes after severe acute malnutrition in Malawian children (ChroSAM): a cohort study

**DOI:** 10.1016/S2214-109X(16)30133-4

**Published:** 2016-07-25

**Authors:** Natasha Lelijveld, Andrew Seal, Jonathan C Wells, Jane Kirkby, Charles Opondo, Emmanuel Chimwezi, James Bunn, Robert Bandsma, Robert S Heyderman, Moffat J Nyirenda, Marko Kerac

**Affiliations:** a Institute for Global Health, University College London, London, UK; b Childhood Nutrition Research Centre, Institute of Child Health, University College London, London, UK; c Respiratory, Critical Care & Anaesthesia section in IIIP, Institute of Child Health, University College London, London, UK; d Division of Infection & Immunity, University College London, London, UK; e Leonard Cheshire Disability & Inclusive Development Centre, Department of Epidemiology & Child Health, University College London, London, UK; f Malawi-Liverpool Wellcome Trust Clinical Research Programme, University of Malawi College of Medicine, Blantyre, Malawi; g Department of Medical Statistics, London School of Hygiene & Tropical Medicine, London, UK; h Department of Population Health, London School of Hygiene & Tropical Medicine, London, UK; i Alder Hey Children's NHS Foundation Trust, Liverpool, UK; j Department of Pediatrics, Division of Gastroenterology, Hepatology and Nutrition, The Hospital for Sick Children, Toronto, ON, Canada

## Abstract

**Background:**

Tackling severe acute malnutrition (SAM) is a global health priority. Heightened risk of non-communicable diseases (NCD) in children exposed to SAM at around 2 years of age is plausible in view of previously described consequences of other early nutritional insults. By applying developmental origins of health and disease (DOHaD) theory to this group, we aimed to explore the long-term effects of SAM.

**Methods:**

We followed up 352 Malawian children (median age 9·3 years) who were still alive following SAM inpatient treatment between July 12, 2006, and March 7, 2007, (median age 24 months) and compared them with 217 sibling controls and 184 age-and-sex matched community controls. Our outcomes of interest were anthropometry, body composition, lung function, physical capacity (hand grip, step test, and physical activity), and blood markers of NCD risk. For comparisons of all outcomes, we used multivariable linear regression, adjusted for age, sex, HIV status, and socioeconomic status. We also adjusted for puberty in the body composition regression model.

**Findings:**

Compared with controls, children who had survived SAM had lower height-for-age *Z* scores (adjusted difference *vs* community controls 0·4, 95% CI 0·6 to 0·2, p=0·001; adjusted difference *vs* sibling controls 0·2, 0·0 to 0·4, p=0·04), although they showed evidence of catch-up growth. These children also had shorter leg length (adjusted difference *vs* community controls 2·0 cm, 1·0 to 3·0, p<0·0001; adjusted difference *vs* sibling controls 1·4 cm, 0·5 to 2·3, p=0·002), smaller mid-upper arm circumference (adjusted difference *vs* community controls 5·6 mm, 1·9 to 9·4, p=0·001; adjusted difference *vs* sibling controls 5·7 mm, 2·3 to 9·1, p=0·02), calf circumference (adjusted difference *vs* community controls 0·49 cm, 0·1 to 0·9, p=0·01; adjusted difference *vs* sibling controls 0·62 cm, 0·2 to 1·0, p=0·001), and hip circumference (adjusted difference *vs* community controls 1·56 cm, 0·5 to 2·7, p=0·01; adjusted difference *vs* sibling controls 1·83 cm, 0·8 to 2·8, p<0·0001), and less lean mass (adjusted difference *vs* community controls −24·5, −43 to −5·5, p=0·01; adjusted difference *vs* sibling controls −11·5, −29 to −6, p=0·19) than did either sibling or community controls. Survivors of SAM had functional deficits consisting of weaker hand grip (adjusted difference *vs* community controls −1·7 kg, 95% CI −2·4 to −0·9, p<0·0001; adjusted difference *vs* sibling controls 1·01 kg, 0·3 to 1·7, p=0·005,)) and fewer minutes completed of an exercise test (sibling odds ratio [OR] 1·59, 95% CI 1·0 to 2·5, p=0·04; community OR 1·59, 95% CI 1·0 to 2·5, p=0·05). We did not detect significant differences between cases and controls in terms of lung function, lipid profile, glucose tolerance, glycated haemoglobin A_1c_, salivary cortisol, sitting height, and head circumference.

**Interpretation:**

Our results suggest that SAM has long-term adverse effects. Survivors show patterns of so-called thrifty growth, which is associated with future cardiovascular and metabolic disease. The evidence of catch-up growth and largely preserved cardiometabolic and pulmonary functions suggest the potential for near-full rehabilitation. Future follow-up should try to establish the effects of puberty and later dietary or social transitions on these parameters, as well as explore how best to optimise recovery and quality of life for survivors.

**Funding:**

The Wellcome Trust.

## Introduction

Tackling severe acute malnutrition (SAM) is a major global health priority, as emphasised by its inclusion in the Sustainable Development Goals (SDGs).^1^ Although SAM-related mortality has been well described,^2^ little is known about its long-term effects on the health or quality of life of survivors. As global child mortality falls, these long-term considerations are becoming increasingly important. Generation Nutrition, a recent major campaign, emphasises this need to look at the long-term health effects of SAM.^3^ For programmes and policies to effectively achieve such goals, further evidence is needed.

One potential long-term outcome for individuals who survive SAM is an increased risk of non-communicable diseases (NCDs) in later life.^4^, ^5^ Extensive evidence has linked variability in early life nutrition with adult health and NCDs:^6^ a topic referred to as developmental origins of health and diseases (DOHaD). Most evidence for DOHaD describes associations between in utero or very early postnatal exposures and adult NCD risk. However, it is biologically plausible that the occurrence of an insult such as SAM during late infancy or early childhood could have similar lasting effects, since crucial growth and development occurs beyond infancy and even into puberty.^7^ Both the physiological insult of an acute, extreme calorie shortage, and the rapid catch-up growth that occurs during and after treatment could have long-term implications. These early life events might contribute to the high burden of deaths caused by cardiovascular disease, diabetes, and lung disease in low-income and middle-income countries (which account for 80% of the 36 million deaths globally per year), necessitating the need for appropriate research.^8^

**Research in context**

**Evidence before this study**

Very few studies have investigated the long-term implications of severe acute malnutrition (SAM). In a 2012 literature review, Bahwere and colleagues searched PubMed and Google Scholar using the terms “protein energy malnutrition”, “protein caloric malnutrition”, “severe malnutrition”, “marasmus”, “kwashiorkor” and “after recovery”, “post-discharge” or “long term”. They found eight follow-up studies describing children between 6 and 18 months after discharge from SAM treatment. The major finding was persistent post-SAM stunting. Although some longer follow-up studies of SAM do exist, they used older case definitions of SAM, have small sample sizes, or focus only on survival and linear growth. By contrast, many studies have conducted long-term follow-up for low birthweight infants and identified an association between early life nutrition and adult health, especially non-communicable diseases (NCDs). This field of research is known as developmental origins of health and disease (DOHaD).

**Added value of this study**

Our study fills an important evidence gap that has emerged since treatment for SAM has improved and SAM-associated mortality has decreased: long-term consequences increasingly matter. Our data following up survivors of SAM 7 years after discharge from hospital covered a wide range of outcomes, as well as recruiting both sibling and community controls. The results help to define a potential further window of plasticity for DOHaD because they describe the effects of a nutritional insult during childhood rather than the fetal or early infant period.

**Implications of all the evidence**

We found that SAM has long-term implications for growth, body composition, and physical function 7 years after hospital discharge. Conversely, sitting height, head circumference, and cardiometabolic and pulmonary markers of NCD risk seem to be largely preserved. Combining evidence from this study with existing evidence for DOHaD suggests that survivors of SAM show traits of so-called thrifty growth. Further studies are needed to establish the full effects of SAM as these individuals undergo puberty and encounter other potential stressors such as increased access to diets high in fat and sugar during ongoing urbanisation of Malawi. Further research is also needed to enable public health programming and policy makers to reduce long-term consequences. It is no longer sufficient for children affected by SAM to just survive: interventions must also help them to thrive.

This study aimed to explore the long-term effects of SAM in children 7 years after they had been discharged from hospital where they were receiving treatment for SAM. We investigated the effects of SAM on growth, body composition, functional outcomes, and risk factors for NCDs in a large nutrition cohort in sub-Saharan Africa.

## Methods

### Study design and participants

The original prospective cohort consisted of 1024 patients admitted for treatment of SAM at the Moyo nutrition ward at Queen Elizabeth Central Hospital in Blantyre, Malawi, from July 12, 2006, to March 7, 2007. All patients were treated in accordance with the national guidelines at the time.^9^ This treatment involved admission based on National Center for Health Statistics (NCHS) references and initial inpatient stabilisation for all children by use of therapeutic milk followed by nutritional rehabilitation at home with ready-to-use therapeutic food. Detailed baseline data were collected as part of the PRONUT study.^10^ Median age at admission was 24 months (IQR 16–34). In a follow-up study at one year post-discharge (FuSAM),^11^ 477 (47%) children from the original cohort remained alive; the surviving children from this study form the case group for the present follow-up, the ChroSAM study. For comparison, we aimed to recruit one sibling control and one community control per child in the case group. The sibling control was the sibling closest in age to the case child, limited to children aged between 4 and 15·9 years. The community control was defined as a child living in the same community, of the same sex, and aged within 12 months of the case child. We selected community controls randomly by spinning a bottle at the case child's home and enquiring door to door, starting from the nearest house to where the bottle pointed. Written informed consent was obtained from the children's parent or guardian. Any children who had ever been treated for SAM were not eligible as controls. Additional assent was sought from the child if they were older than 13 years. Ethical approval for the study was granted by the Malawi College of Medicine Research and Ethics Committee (reference P.02/13/1342), and the University College London Research Ethics Committee (reference 4683/001).

### Outcomes

Our outcomes of interest were anthropometry, body composition, lung function, physical capacity (ie, hand grip strength, step test, and physical activity), school achievement, and blood markers of NCD risk. Exposures included previous admission for SAM, socioeconomic status, HIV, and maternal education.

Anthropometric assessments were done in accordance with the guidelines by Lohman and colleagues^12^ and WHO^13^ and were subject to quality control, which involved two members of the trained study team taking independent readings.^13^ Body composition was measured with skinfold thickness measured at the biceps, triceps, subscapular, and suprailiac sites, and by use of bioelectrical impedance analysis (BIA) with a Quadscan 4000 device (Bodystat, Douglas, Isle of Man). Usually, BIA outputs are converted to total body water and fat-free mass via population-specific empirical equations.^14^ In the absence of a population-specific equation, it is possible to assess relative hydration and lean mass by use of raw BIA values adjusted for height.^15^ Results are presented as resistance index (R/height) and reactance index (Xc/height).

Physical activity was measured in a subset (n=78) of children with Actilife GT3X accelerometers (ActiGraph, Pensacola, FL, USA). Muscle strength was measured with a Takei Grip-D device (Takei, Niigata, Japan). Physical capacity was measured with the iSTEP (incremental step) test.^16^ Lung function was measured with spirometry on an Easy-On PC device (ndd Medical Technologies, Zürich, Switzerland); quality grades were applied in a blinded manner by a senior respiratory physiologist at the Institute for Child Health of University College London (London, UK).^17^ Spirometry outcomes were forced expiratory volume in 1 second (FEV_1_), forced vital capacity (FVC), and the FEV_1_-to-FVC ratio, all expressed as *Z* scores.^18^ More detailed methods are available in the appendix (pp 9–11).

Venous blood samples to measure glycated haemoglobin (HbA_1c_), lipid profile, and full blood count were taken after 12 h of fasting. Salivary cortisol was assessed about mid-morning. A subset of children (n=60) underwent an oral glucose tolerance test with 1·75 mg per kg bodyweight of Polycal glucose powder (Nutricia, Dublin, Ireland).^19^

Data collectors were not blinded to the case or control status of the children because of the logistics of the study. The HIV status of children and mothers was established from health passports; if status was not known or if the test was done before the age of 18 months, HIV testing was offered by a trained counsellor. Puberty was recorded as a binary variable, as reported by the participant or guardian (onset of menarche in girls, voice change in boys). Socioeconomic status was derived from asset scores calculated with questions taken from the Malawi Demographic Health Survey.^20^

### Statistical analysis

Sample size was predetermined by the cohort size and survival. Controls were more difficult to recruit than were cases because they had no previous personal connection with the study team and were restricted to the number of eligible children in the family and community. However, with 320 cases, 217 sibling controls, and 184 community controls, we calculated that the study had a post-hoc power of at least 90% to detect a *Z* score difference of 0·5 between the cases and controls based on reference data for growth and lung function outcomes at the 5% level of statistical significance. Given that *Z* scores of 0·5 are considered clinically significant in lung function testing and that the FuSAM study showed a difference in weight-for-age *Z* score (WAZ) of 0·55 and height-for-age *Z* score (HAZ) of 1·13 between cases and sibling controls,^11^ we deemed our sample size to be satisfactory. Post-hoc calculations suggested that the sample size was adequate for all outcomes except physical activity (steps per day), which was underpowered.

We did statistical analyses with Stata version 12.1. To calculate the WAZ, HAZ, and body-mass index (BMI)-for-age *Z* score (BAZ), we used WHO AnthroPlus, which includes new WHO reference curves for children aged 5–19 years.^21^ We analysed BIA data with BIA vector analysis, which graphically illustrates the association between resistance and reactance measures (phase angle) to give an indication of variability in hydration versus lean mass.^22^ We converted spirometry outcomes to *Z* scores with the Global Lung Function Initiative (GLI) African-American reference values, which adjust for height, age, sex, and ethnicity.^18^

In the main analysis, we compared each type of control with case children by use of simple and multivariable linear regression analysis. We included age, sex, HIV status, and socioeconomic status as potential confounders in all multivariable regressions. We also included puberty as a potential confounder for body composition; puberty and sitting height were additional potential confounders for lung function outcomes. We used ordered logistic regression to analyse differences between cases and controls for time completed in the exercise test and school grade achieved, which we used as a measure of educational attainment since children in Malawi do not move to the next grade until they pass, irrespective of their age. Because anthropometry was collected for some sibling controls during the 1-year follow-up study,^11^ we used repeated measures mixed regression models to analyse longitudinal growth between cases and sibling controls between 1 and 7 years post-discharge. In all analyses, we deemed a p value of less than 0·05 as showing a statistically significant difference between groups.

### Role of the funding source

The funder of the study had no role in study design, data collection, data analysis, data interpretation, or writing of the report. The corresponding author had full access to all the data in the study and had final responsibility for the decision to submit for publication.

## Results

**Figure 1:**
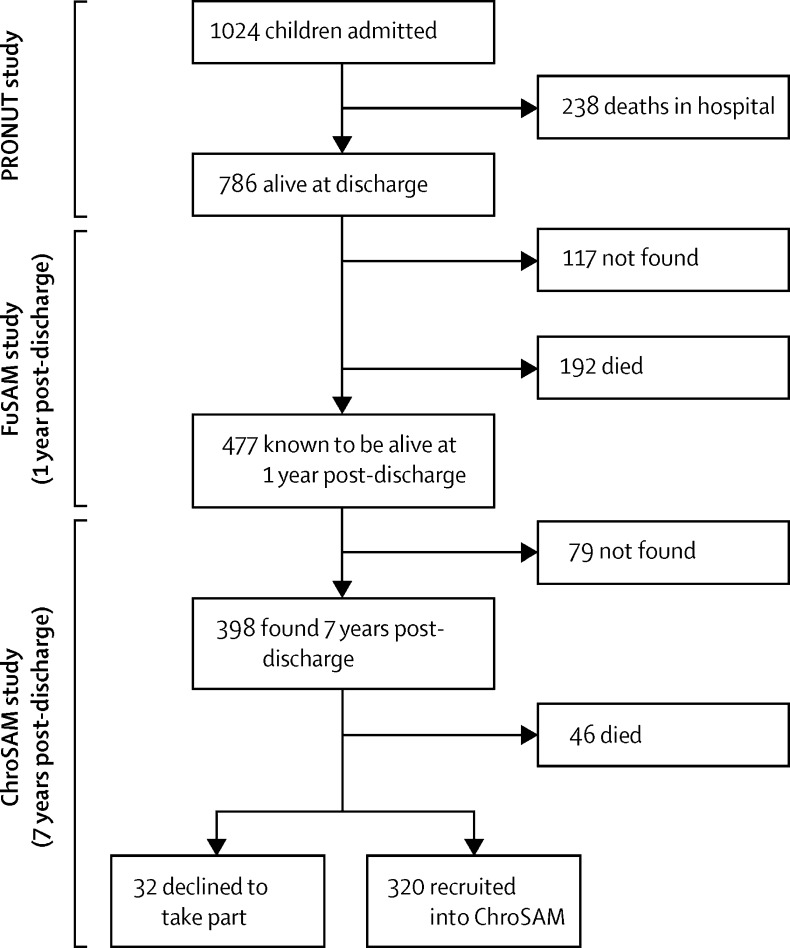
Recruitment of the case group Flow diagram for showing recruitment, starting with original recruitment in 2006 for the PRONUT study,^10^ followed by 1-year follow up in the FuSAM study,^11^ and the present follow-up (ChroSAM).

**Figure 2:**
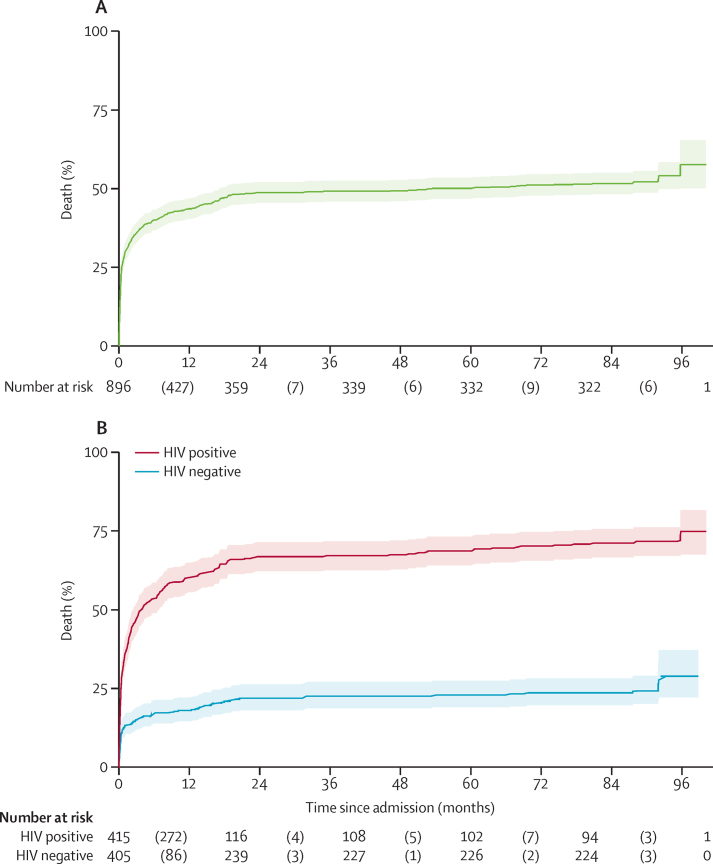
Time-to-death analysis Kaplan-Meier time-to-death curve showing probability of death for all children in the case group (A) and probability of death for children stratified by HIV serostatus (B). Numbers in parentheses are numbers of deaths.

Figure 1 shows the recruitment of case children; sample sizes differ slightly for individual outcomes, mostly because not everyone who was recruited and assessed in the field came for a hospital visit. Between 1 year and 7 years post-discharge, 46 (12%) of the 398 children who could be found after 7 years had died; of the 46 children who died during this period, 25 (54%) were HIV seropositive, 10 (22%) had a clinically obvious disability (eg, cerebral palsy), and 11 (24%) had neither HIV nor disability. Figure 2 shows Kaplan-Meier time-to-death curves that are an extension of those reported previously in the 1 year post-discharge study.^11^ These curves show that most deaths occurred within 2 years after admission (figure 2A) and that HIV had a substantial effect on survival (figure 2B). A possible explanation for the apparent step-up in deaths at about 90 months (7 years) post-admission is recall bias with respect to date of death. The mean mortality rate between 2008 and 2013 for children aged 5–9 years in this cohort was 0·014 deaths per 1000 person-years, which is high compared with the normal rate for this age group in Malawi of 0·003 deaths per 1000 person-years, as reported in the WHO life table.^23^

Table 1 shows health and demographic characteristics of the three study groups. Characteristics of children in the case group who have died and who were lost to follow-up are given in the appendix (p 1). Children lost to follow-up had similar basic demographics and clinical history to those in the case group in this study (appendix p 1).

In terms of HAZ, case children had more severe stunting than controls, with shorter leg length (table 2) but similar sitting height (results for HIV-negative children only are given in the appendix p 3). Community and sibling controls were more likely to be in a higher school grade than were cases after adjustment for age, sex, HIV status, and socioeconomic status (odds ratio [OR] 1·70, 95% CI 1·2–2·4, p=0·003 compared with community controls and OR 2·77, 95% CI 1·9–4·0, p<0·0001 compared with sibling controls).

**Figure 3:**
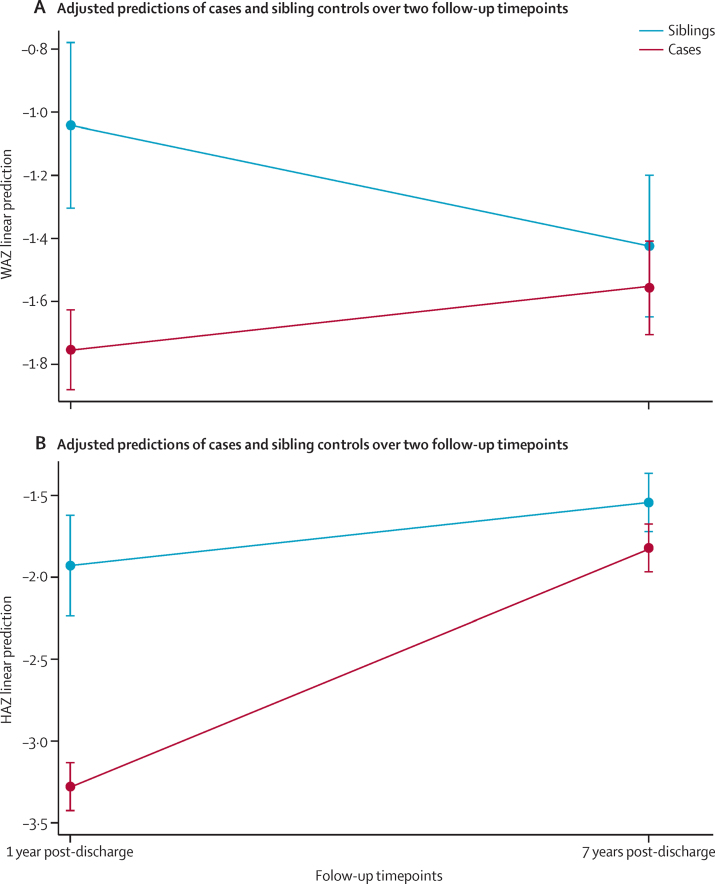
Changes in anthropometry Graphs show modelled means for WAZ (A) and HAZ (B) for siblings and cases at 1 and 7 years post-discharge. WAZ=weight-for-age *Z* score. HAZ=height-for-age *Z* score.

In our subgroup analysis of longitudinal growth in cases and sibling controls between 1 and 7 years post-discharge, cases showed a significant increase in HAZ (coefficient 1·45, p<0·0001) over time after adjustment for age, sex, and HIV status. Compared with sibling controls, cases had larger changes in HAZ (difference 1·1, p<0·0001) and WAZ (difference 0·9, p<0·0001), suggesting that cases were catching up their lost WAZ and HAZ by growing at a faster rate than were sibling controls (figure 3).

In our assessment of body composition (table 2), cases had smaller calf and mid-upper arm circumferences (MUAC), suggesting reduced peripheral mass compared with controls. Cases had smaller hip circumference compared with controls and larger or similar waist circumference, suggesting an unhealthy ratio of core to gluteofemoral fat. In the BIA vector analysis, cases had significantly less lean mass than did community controls, but similar levels to siblings after adjustment for age differences (appendix p 12). Cases also had lower phase angles than did community controls, which is associated with risk of malnutrition and morbidity risk.^24^

Cases had significantly weaker hand grip strength than did community controls (table 3). Results for HIV-negative children only are presented in the appendix (p 4). Cases took on average 239 fewer steps per hour than did their sibling controls and 54 fewer steps per hour than did community controls; however, these differences were not significant. Additionally, both sibling and community controls had greater odds of completing more minutes of the iStep exercise test than did cases (sibling OR 1·59, 95% CI 1·0–2·5, p=0·04; community OR 1·59, 95% CI 1·0–2·5, p=0·05).

Most of the other NCD risk factors that we assessed were not significantly different between cases and controls (table 3), except for diastolic blood pressure, which was higher for cases than for sibling controls (adjusted difference 1·91 mm Hg, p=0·03). In the oral glucose tolerance test, three (5%) of 56 children had impaired fasting glycaemia and three (5%) others had impaired glucose tolerance; there were no differences between the groups when compared with Fisher's exact test (cases *vs* sibling controls, p=0·20; cases *vs* community controls, p=1·0).

## Discussion

A follow-up study of this size and length in a setting where SAM is prevalent is novel,^25^ as is the breadth of outcomes investigated. Our results suggest that SAM has long-term adverse effects, especially with respect to growth and body composition. Survivors of SAM continue to have significantly more stunting than their siblings and other children in their community at 7 years post-discharge from inpatient nutritional treatment. Associated functional impairments include poorer physical strength, poorer physical capacity, and lower school achievement than in controls. Our results could inform future interventions to address the complex links between wasting and stunting, which are increasingly being debated.^26^

Despite greater stunting in the case group than in controls, sitting height was similar across the groups, suggesting that torso growth has been preserved and limb growth compromised. Additionally, head circumference was similar in all groups and lung function and HbA_1c_ were close to normal when compared with black American children in all groups.^18^, ^27^ Total cholesterol was also within UK recommendations. Survivors of SAM might have undergone so-called brain-sparing—or thrifty—growth, whereby the growth of vital organs has been preserved at the cost of less vital growth.^28^, ^29^

Although body composition and physical function outcomes suggest potentially greater risk of NCDs in later life,^30^, ^31^ at the relatively early life stage studied, we found little evidence of impaired respiratory or cardiometabolic outcomes (apart from increased diastolic blood pressure). Large sitting-to-standing height ratio, short limb length, lower peripheral mass, and larger waist-to-hip ratio have all been associated with NCDs in adulthood, such as cardiovascular disease, diabetes, and hypertension.^30^, ^32^, ^33^, ^34^ Peripheral fat and lean mass, especially in terms of greater hip circumference, are thought to be protective against cardiovascular disease.^30^ The pattern of lower lean mass (after adjusting for height) and preservation of fat mass seen in children in the case group is similar to that seen in children born with low birthweight^35^ and is an important predictor of physical work capacity in later life.^36^ The combination of reduced lean mass and greater stunting compared with controls might explain the deficiencies in physical function and strength seen in the survivors of SAM in our study.^36^, ^37^ Lean mass, glucose tolerance, and activity levels are interlinked and have implications for later-life NCDs and earning potential.^38^, ^39^ Weaker hand grip is also associated with lower bone mass, impaired cell membrane potential, and reduced muscle function, as well as all-cause early mortality, risk of malnutrition, and risk of NCDs.^40^

However, the children in the case group did show signs of growth recovery. Although gain in HAZ was minimal in the first year post-discharge,^11^ it has since increased. Comparing the growth of cases with that of sibling controls between 1 and 7 years post-discharge, we noted that HAZ was increasing at a steeper rate in cases, who were catching up to their sibling controls. There has been much conflicting data about the possibility of catch-up growth in children with stunting, especially after the first 1000 days of life.^41^, ^42^ In one study, the authors noted that both growth faltering and catch-up growth could occur between the ages of 8 years and 15 years, concluding that this period offers continued opportunities and sustained risks for children.^43^ Our results concur with this study, suggesting that these survivors of SAM are on track to catch up some of their lost linear growth. Since their growth plates have not yet fused, it would be interesting to assess stunting and sitting height ratio after adolescence.^7^ Assessment of whether gains in height translate to functional improvements, such as improved school achievement,^44^ will also be necessary.

The potential for catch-up growth, as well as the apparent preservation of vital organ functions shown by our spirometry and cardiometabolic results, suggests that there might be potential for recovery following SAM. Better follow-up care and post-SAM interventions might reverse stunting, build lost lean mass, and improve muscle strength and physical activity levels in SAM survivors, thereby improving their quality of life and reducing their risk of NCDs in later life. However, it is notable that these SAM survivors remain relatively small compared with the global population for this age (largest WAZ being only 0·48) and disrupted organ function might only become apparent when exposed to increased weight and unhealthy lifestyles,^45^ as seen in studies of low birthweight.^46^ With the changes in dietary trends occurring in many African countries,^47^ it is possible that, in the future, SAM survivors will face increasing weight gain and therefore potentially greater NCD risks.

Survivor bias is the most important limitation of our study given that only 352 of 1024 children in the original cohort were still alive for the present follow-up. Consequently, our detection of impairments in these children, the fittest and healthiest survivors, is of particular concern. As general and SAM-associated mortality fall, and less healthy children also survive, the long-term consequences might become even more marked.

Second, our results might not be generalisable to all children currently being treated for SAM. Although the children in our study were all treated as inpatients according to NCHS admission criteria, the latest treatment protocols focus on outpatient care (community-based management of acute malnutrition [CMAM]) wherever possible,^48^ and admission criteria are also now more inclusive.^49^ Because CMAM includes earlier treatment and slower weight gain, future survivors might have fewer long-term effects. Conversely, because CMAM enables a better survival rate, it could lead to more long-term adverse effects because the most affected children who would previously have died are now surviving. Long-term follow-up of a cohort treated with CMAM is needed to establish which effects dominate.

Third, sibling and community controls are not fully healthy. These children also have stunting although not as severe as in the case group. The controls are exposed to similar socioeconomic constraints to the case group and have as many hospital admissions. As the general health of the population improves, the effects of SAM may be more noticeable.

Fourth, it is important to note that, in this observational study, we cannot distinguish between association and causality. For example, we do not know to what extent early stunting contributed to episodes of SAM or whether SAM itself provokes greater stunting.^26^, ^50^

Finally, we do not have data on early life demographics, including exact birthweight and gestational age. These factors could be potential confounders, since they are linked to both SAM and long-term adverse outcomes.

We have identified enduring effects of SAM on growth, body composition, and physical function, even in a diminished group of survivors. The absence of cardiometabolic or pulmonary markers of NCD risk in these survivors, as well as the presence of catch-up growth, suggest the potential for recovery with appropriate intervention following SAM. However, further follow-up is needed to establish the full effects of SAM after puberty, including the additional effects of urbanisation and access to diets high in fat and sugar. Interventional studies to explore earlier treatment and improved post-discharge care are a priority so that future policy and programming can minimise any long-term adverse outcomes and help children who survive SAM to thrive.

## Figures and Tables

**Table 1:** Demographic and health characteristics

		**Cases (n=320)**	**Sibling control (n=217)**	**Community control (n=184)**
**Basic demographics**				
Age (years)		9·3 (8·1–10·3)	10·9 (7·4–12·8)	9·1 (8·3–10·2)
Males		174 (54%)	106 (49%)	96 (52%)
Birth order		2 (1–4)	2 (2–3)	2 (1–3)
Started puberty		7/320 (2%)	14/215 (7%)	6/180 (3%)
**Clinical history**				
HIV				
	Seropositive	90/320 (28%)	9/217 (4%)	5/184 (3%)
	Seronegative	208/320 (65%)	130/217 (60%)	95/184 (52%)
	Status unknown	22/320 (7%)	78/217 (36%)	84/184 (46%)
Visited outpatient clinic in past 6 months		115/315 (37%)	56/214 (26%)	51/179 (28%)
**Family**				
Mother died		51/303 (17%)	..	4/160 (3%)
Father died		59/286 (21%)	..	9/151 (6%)
Mother HIV positive		83/222 (37%)	..	23/135 (17%)
Mother's age at time of ChroSAM (years)		32 (18–62)	..	30 (19–50)
Maternal height (cm)		156·9 (5·7)	..	156·4 (5·9)
Parity		3 (3–5)	..	4 (3–5)
**Home environment**			**..**	
Unimproved toilets*		244/315 (77%)	..	140/173 (81%)
Cooking inside with solid smoke†		47/317 (15%)	..	21/174 (12%)
**Socioeconomic status**				
Maternal education				
	None	56/309 (18%)	..	22/173 (13%)
	Primary	121/309 (39%)	..	65/173 (38%)
	Secondary	132/309 (43%)	..	86/173 (50%)
Wealth asset quintile				
	1 (poorest)	67/311 (22%)	..	30/174 (17%)
	2	59/311 (19%)	..	41/174 (24%)
	3	61/311 (20%)	..	28/174 (16%)
	4	61/311 (20%)	..	36/174 (21%)
	5 (richest)	63/311 (20%)	..	39/174 (22%)

Data are median (IQR), mean (SD), n (%) or n/N (%). Some characteristics have different denominators due to missing data.

* Open pit latrine, field, or bush.

† Cooking with wood or charcoal inside the home.

**Table 2:** Differences in growth and body composition between cases and controls

	**Cases (n=378)**	**Sibling controls (n=219)**	**Cases *vs* sibling controls**		**Community controls (n=184)**	**Cases *vs* community controls**	
			Adjusted difference (95% CI)	p value		Adjusted difference (95% CI)	p value
**Growth indices**							
WAZ	−1·6 (0·9)	−1·4 (1·0)	−0·2 (−0·5 to 0·1)	0·16	−1·2 (0·9)	0·3 (−0·0 to 0·5)	0·06
BAZ	−0·8 (0·9)	−0·8 (0·9)	0·08 (−0·1 to 0·3)	0·39	−0·7 (0·9)	0·1 (−0·1 to 0·3)	0·31
HAZ	−1·8 (1·2)	−1·5 (1·2)	0·2 (0·0 to 0·4)	0·04	−1·3 (1·1)	0·4 (0·2 to 0·6)	0·001
**Height**							
Standing (cm)	124·9 (9·0)	130·3 (16·8)	2·0 (0·6– 3·4)	0·004	127·4 (9·9)	2·7 (1·2 to 4·2)	0·001
Sitting (cm)	65·2 (4·4)	67·4 (7·6)	0·6 (−0·1 to 1·3)	0·07	65·9 (4·5)	0·7 (−0·1 to 1·5)	0·08
Leg length (cm)	59·9 (5·5)	63·0 (9·6)	1·4 (0·5 to 2·3)	0·002	61·6 (6·0)	2·0 (1·0 to 3·0)	<0·0001
**Circumferences**							
Head (cm)	51·1 (2·1)	52·1 (2·5)	0·1 (−0·3 to 0·5)	0·31	52·1 (1·9)	0·3 (−0·1 to 0·8)	0·12
MUAC (mm)	172 (20)	183 (29·8)	5·7 (2·3 to 9·1)	0·002	178 (22)	5·6 (1·9 to 9·4)	0·001
Calf (cm)	23·7 (2·3)	25·0 (3·5)	0·62 (0·2 to 1·0)	0·001	24·3 (2·4)	0·49 (0·1 to 0·9)	0·01
**Body composition**							
R/height	603·5 (105·1)	567·4 (130)	−11·5 (−29 to −6)	0·19	577·1 (90·7)	−24·5 (−43 to −5·5)	0·01
Xc/height	52·2 (8·4)	49·7 (9·3)	−0·5 (−2·1 to 1·0)	0·52	51·5 (7·8)	0·3 (−1·4 to 1·9)	0·75
Phase angle (°)	5·0 (0·7)	5·1 (0·7)	0·1 (−0·0 to 0·2)	0·16	5·1 (0·5)	0·2 (0·1 to 0·3)	0·004
Waist circumference (cm)	56·3 (4·2)	57·7 (6·4)	0·55 (−0·2 to 1·3)	0·15	56·0 (4·5)	0·08 (−0·7 to 0·9)	0·86
Hip circumference (cm)	62·3 (5·8)	65·9 (10·2)	1·83 (0·8 to 2·8)	<0·0001	63·7 (6·9)	1·56 (0·5 to 2·7)	0·01
Waist-to-hip ratio	0·91 (0·1)	0·88 (0·1)	−0·01 (−0·0 to 0)	0·07	0·89 (0·1)	−0·02 (−0·0 to −0·0)	0·01

Data are mean (SD) unless noted otherwise. Adjusted differences are the results of multivariable linear regression, adjusted for age, sex, HIV status, and socioeconomic status for all outcomes. The body composition regression model was also adjusted for puberty. Unadjusted results are presented in the appendix (pp 5–6). WAZ=weight-for-age *Z* score. BAZ=body-mass-index-for-age *Z* score. HAZ=height-for-age *Z* score. MUAC=mid-upper arm circumference. R/height=resistance index. Xc/height=reactance index.

**Table 3:** Means and differences in measures of NCD risk between cases and controls

	**Cases (n=378)**	**Sibling controls (n=219)**	**Cases *vs* sibling controls**		**Community controls (n=184)**	**Cases *vs* community controls**	
			Adjusted difference (95% CI)	p value		Adjusted difference (95% CI)	p value
**Cardiorespiratory function**							
FEV1 *Z* score	−0·47 (1·1)	−0·48 (1·0)	−0·02 (−0·3 to 0·2)	0·88	−0·34 (1·1)	0·10 (−0·2 to 0·4)	0·46
FVC *Z* score	−0·32 (1·0)	−0·38 (1·1)	−0·11 (−0·4 to 0·1)	0·41	−0·15 (1·1)	0·12 (−0·1 to 0·1)	0·36
FEV1/FVC ratio *Z* score	−0·21 (0·9)	−0·15 (0·9)	0·14 (−0·1 to 0·4)	0·25	−0·37 (1·0)	−0·13 (−0·4 to 0·1)	0·32
Systolic blood pressure (mm Hg)	107·8 (9·5)	109·5 (11·4)	−0·17 (−2·0 to 1·6)	0·86	108·3 (9·4)	0·17 (−1·8 to 2·2)	0·87
Diastolic blood pressure (mm Hg)	69·9 (8·6)	68·8 (9·6)	−1·91 (−3·6 to −0·2)	0·03	68·4 (9·0)	−1·60 (−3·5 to 0·3)	0·10
**Physical function**							
Hand grip strength (kg)	12·7 (6·3)	14·8 (7·9)	1·01 (0·3 to 1·7)	0·005	13·8 (3·9)	1·68 (0·9 to 2·4)	<0·0001
Steps per hour (n=78)	716·5 (413)	955·8 (361)	144·5 (−37 to 325)	0·12	770·3 (367)	−6·72 (−185 to 172)	0·94
**Metabolic status**							
Total cholesterol (mmol/L)	3·23 (0·9)	3·29 (1·0)	0·02 (−0·2 to 0·2)	0·81	3·19 (0·8)	−0·08 (−0·3 to 0·1)	0·46
Total cholesterol-to-high density lipoprotein ratio	3·69 (1·5)	3·87 (1·9)	−0·04 (−0·4 to 0·3)	0·81	3·82 (1·4)	−0·13 (−0·5 to 0·3)	0·51
Baseline glucose (n=59)	4·54 (0·6)	4·81 (0·8)	0·59 (0·1 to 1·1)	0·02	4·71 (0·4)	0·25 (−0·5 to 1·0)	0·52
120 min glucose (n=56)	5·54 (1·3)	5·93 (0·9)	−0·20 (−1·1 to 0·7)	0·65	6·21 (1·5)	0·13 (−1·3 to 1·6)	0·86
Salivary cortisol (nmol/L; n=82)	4·49 (1·7)	4·73 (1·8)	0·32 (−0·7 to 1·3)	0·53	5·08 (1·7)	0·77 (−0·6 to 2·1)	0·26
Glycated haemoglobin A_1c_ (%)	5·13 (0·5)	5·17 (0·5)	0·05 (−0·1 to 0·2)	0·40	5·07 (0·5)	−0·06 (−0·2 to 0·1)	0·36

Data are mean (SD) unless noted otherwise. Adjusted differences are adjusted for age, sex, HIV status, and socioeconomic status in a linear regression model. Puberty and sitting height (%) are included as potential confounders for spirometry outcomes. Steps per hour, glucose tolerance, and salivary cortisol were investigated in a subsample with size (n) indicated. Overall fail rate (grade F) for spirometry results was 12%. Unadjusted results are presented in the appendix (pp 7–8). NCD=non-communicable disease. FEV_1_=forced expiratory volume in 1 s. FVC=forced vital capacity.
