# Supplementary material for: Chronic disease outcomes after severe acute malnutrition in Malawian children (ChroSAM): a cohort study
Source: Lancet Glob Health. 2016 Jul 25;4(9):e654–62. doi: 10.1016/S2214-109X(16)30133-4 (PMC4985564; doi:10.1016/S2214-109X(16)30133-4)
Supplement: Supplementary appendix [file mmc1.pdf]

THE LANCET Global Health

Supplementary appendix

This appendix formed part of the original submission and has been peer reviewed. We post it as supplied by the authors.

Supplement to: Lelijveld N, Seal A, Wells JC, et al. Chronic disease outcomes after severe acute malnutrition in Malawian children (ChroSAM): a cohort study. *Lancet Glob Health* 2016; published online July 25. http://dx.doi.org/10.1016/S2214-109X(16)30133-4.

Supporting Information: Web Annexes

Web Annex Table 1: expanded version of demographic and health characteristics of the 3 study groups.

	**Cases (n=320)**	**ChroSAM deaths (n=46)**	**Sibling Control (n=217)**	**Community control (n=184)**	**Cases Loss to Follow Up (190)**
Basic Demographics					
**Median age /would be age (years)**	9.3 (range 7.4 – 20.1)	10 (range 8.1 – 16)	10.9 (range 4 – 15.6)	9.1 (range 5.1 – 15.1)	8 (range 7 - 19)
**Males (gender)**	174 (55%)	28 (57%)	106 (49%)	96 (52%)	108 (57%)
**Median Birth Order**	2 (IQR 1-4)	3 (IQR 1-9)	2 (IQR 2-3)	2 (IQR 1-3)	2 (IQR 1-3)
Clinical History					
**HIV**					
**Seropositive**	90/320 (28%)	25/46(53%)	9/217 (4%)	5/184 (3%)	44/190(23%)
**Seronegative**	208/320(65%)	20/46 (43%)	130/217(60%)	95/184 (52%)	121/190(64%)
**Unknown**	22/320 (7%)	1/46 (2%)	78/217 (36%)	84/184 (46%)	25/190 (13%)
**History of TB**	18/317 (5.7%)	4/38 (11%)	1/215 (0.5%)	1/179 (0.6%)	4/173 (2%)
**History of pneumonia admissions**	10/317 (3%)	///	15/213 (7%)	9/180 (5%)	///
**Ever admitted to hospital (except SAM)**	70/318 (22%)	///	53/213 (25%)	54/180 (30%)	///
**Visited outpatient clinic in past 6 months**	115/315 (37%)	///	56/214 (26%)	51/179 (28%)	///
Education					
**School grade achieved (median)**	2 (IQR 2-3)	///	3 (IQR 1-5)	3 (IQR 2-4)	///
**Didn't attended school this year(>5.9yrs)**	21/315 (7%)	///	18/196 (9%)	9/176 (5%)	///
Family					
**Mother died**	51/303 (17%)	11/30 (37%)	///	4/160 (3%)	8/159 (5%)
**Mother HIV positive**	83/222 (37%)	10/20 (50%)	///	23/135 (17%)	23/81 (28%)
**Mother's age (median)**	32 (range 18-62)	34 (range 28-47)	///	30 (range 19-50	///
**Maternal Height cm (mean) (SD)**	156.9 (5.7)	155.5 (3.1)	///	156.4 (5.9)	///
**Mother's BMI (mean) (SD)**	22.9 (4.1)	21.8 (2.3)	///	23.6 (4.3)	///
**Maternal MUAC mm (mean) (SD)**	271 (39)	260 (21)	///	282 (41)	///
**Parity~ (median)**	3 (IQR 3-5)	3 (IQR 2-4)	///	4 (IQR 3-5)	///
**Mothers victims of domestic violence**	58/257 (23%)	0/9 (0%)	///	46/135 (34%)	///
**Poor maternal mental health**	16/275 (6%)	5/20 (25%)	///	7/155 (5%)	///
Home environment					
**Exposed to tobacco smoke**	47/317 (15%)	5/35 (14%)	///	30/174 (17%)	///
**Cooking with solid fuel in the**	47/317 (15%)	2/35 (6%)	///	21/174 (12%)	///

**home ***					
**Unimproved toilet Φ**	244/315 (77%)	20/24 (83%)	///	140/173 (81%)	///
Socioeconomic status					
**Mother education**					
**None**	56/309 (18%)	5/14 (35%)	///	22/173 (13%)	13/155 (8%)
**Primary**	121/309(39%)	6/14 (43%)	///	65/173 (38%)	108/155(70%)
**Secondary**	132/309(43%)	3/14 (21%)	///	86/173 (50%)	34/155 (22%)
**Father with no education**	39/270 (14%)	3/13 (23%)	///	11/150 (7%)	5/122 (4%)
**Father unemployed**	120/255(47%)	6/9 (67%)	///	62/158 (39%)	///
**Lowest wealth asset quintile**	67/311 (22%)	3/24 (13%)	48/215 (22%)	30/174 (17%)	///

Web Annex Table 2: Results of regression analysis for growth and body composition outcomes for HIV negative children only

**Anthropometry**	**HIV- Cases (n=228)**	**HIV- Sibling (n=206)**			**HIV- Community (n=175)**		
	Mean (SD)	Mean (SD)	Unadjusted difference (95% CI)	Adjusted difference (95% CI)	Mean (SD)	Unadjusted difference (95% CI)	Adjusted difference (95% CI)
Growth							
**WAZ**	-1.5 (0.9)	-1.4 (1.0)	0.1 (-0.1, 0.4)	-0.2 (-0.5, 0.1)	-1.2 (1.0)	0.3 * (0.1, 0.5)	0.2 * (0.0, 0.5)
**HAZ**	-1.7 (1.2)	-1.5 (1.2)	0.2 (-0.0, 0.4)	0.2 * (0.0, 0.5)	-1.3 (1.1)	0.4 * (0.2, 0.7)	0.4 * (0.1, 0.6)
**BAZ**	-0.8 (1,0)	-0.8 (0.9)	0.03 (-0.2, 0.2)	0.08 (-0.1, 0.2)	-0.7 (0.9)	0.14 (-0.1, 0.3)	0.11 (-0.1, 0.3)
**Standing height (cm)**	125 (8.7)	130.7 (16.8)	5.5 * (3.0, 7.9)	2.1 * (0.7, 3.5)	127.4 (10.1)	2.2 (-0.4, 4.7)	2.5 * (1.0, 4.0)
**Sitting height (cm)**	65.3 (4.3)	67.6 (7.6)	2.3 * (1.1, 3.4)	0.8 * (0.0, 1.5)	66.0 (4.6)	0.7 (-0.5,1.9)	0.8 * (0.0, 1.6)
**Leg length (cm)**	60 (5.4)	63.2 (9.7)	3.2 * (1.7, 4.6)	1.4 * (0.4, 2.3)	61.5 (6.0)	1.5 (-0.1, 3.0)	1.7 * (0.7, 2.7)
**Head circumference (cm)**	51.8 (1.9)	52.2 (2.2)	0.4 * (0.0, 0.8)	0.2 (-0.1, 0.6)	52.1 (1.9)	0.3 (-0.1, 0.7)	0.4 * (0.0, 0.7)
Body composition							
**1/z (lean mass)**	9.07 (1.1)	9.22 (1.0)	0.15 (-0.1,0.3)	0.22 * (0.02, 0.4)	9.13 (1.0)	0.06 (-0.2, 0.3)	0.07 (-0.1,0.3)
**BMI residual from 1/Z (fat mass)**	-0.20 (1.2)	0.98 (1.8)	0.49 * (0.2, 0.8)	0.18 (-0.1, 0.4)	-0.09 (1.6)	0.12 (-0.2, 0.4)	0.12 (-0.2, 0.4)
**r/h**	599 (100)	563 (128)	-36.3 * (-58, -15)	-16.7 (-34, 0.3)	577 (91.8)	-21.9 (-45, 1.1)	-24.5 * (-43,-6.5)
**xc/h**	51.3 (7.8)	49.3 (9.1)	-2.1 * (-3.7,-0.4)	-1.1 (-2.7, 0.4)	51.5 (7.8)	0.19 (-1.6, 2.0)	0.06 (-1.5, 1.7)
**Phase angle ^{0}**	4.9 (0.6)	5.1 (0.7)	0.14 * (0.0,0.3)	0.07 (-0.0, 0.2)	5.1 (0.5)	0.17 * (0.0,0.3)	0.18 * (0.1, 0.3)
**Skinfold thickness ratio**	1.7 (0.4)	1.8 (0.4)	0.07 (-0.0, 0.1)	0.04 (-0.0, 0.1)	1.7 (0.4)	0.03 (-0.1, 0.1)	0.03 (-0.1, 0.1)
**Waist/hip ratio**	0.90 (0.1)	0.88 (0.1)	-0.02 * (-0.03, -0.0)	-0.01 (-0.02, 0.0)	0.89 (0.1)	-0.02 * (-0.03, -0.0)	-0.02 * (-0.0,-0.0)

Adjusted difference includes age, sex and SES in regression model. Body composition outcomes also have puberty included as a potential confounder in adjusted difference

Web Annex Table 3: Results of regression analysis for NCD risk outcomes for HIV negative children only

Outcomes	HIV- Cases (n=228)	HIV- Sibling (n=206)			HIV- Community (n=175)		
	Mean (SD)	Mean (SD)	Unadjusted difference (95% CI)	Adjusted difference (95% CI)	Mean (SD)	Unadjusted difference (95% CI)	Adjusted difference (95% CI)
Cardiorespiratory Function							
**FEV_1 Z score**	-0.32 (1.0)	-0.51 (1.0)	-0.18 (-0.4, 0.1)	-0.19 (-0.4, 0.1)	-0.31 (1.1)	0.01 (-0.2, 0.3)	-0.02 (-0.3, 0.2)
**FVC Z score**	-0.19 (0.9)	-0.41 (1.0)	-0.22 (-0.5, 0.0)	-0.26 * (-0.5, -0.0)	-0.13 (1.0)	0.06 (-0.2, 0.3)	0.03 (-0.2, 0.3)
**FEV_1:FVC Z score**	-0.16 (1.0)	-0.14 (0.9)	0.02 (-0.2, 0.3)	0.10 (-0.1, 0.3)	-0.34 (1.0)	-0.18 (-0.4, 0.1)	-0.18 (-0.4, 0.1)
**Systolic blood pressure**	108.0 (9.2)	110.0 (11.4)	1.81 (-0.2, 3.8)	0.30 (-1.5, 2.1)	108.3 (9.4)	0.36 (-1.8, 2.5)	0.60 (-1.3, 2.5)
**Diastolic blood pressure**	69.8 (8.5)	68.8 (9.6)	-1.01 (-2.8, 0.8)	-1.75 * (-3.5,-0.0)	68.4 (9.0)	-1.45 (-3.4, 0.5)	-1.40 (-3.3, 0.5)
Physical function							
**Hand grip strength**	12.3 (3.8)	14.6 (6.8)	2.26 * (1.3, 3.3)	1.03 * (0.3, 1.7)	13.8 (3.9)	1.47 * (0.4, 2.5)	1.66 * (0.9, 2.4)
**Steps per hour (n=63)**	839.9 (292)	1017 (296)	177.5 * (5.5, 350)	202.3 * (17,388)	860.6 (213)	20.76 (-143, 184)	60.84 (-122, 234)
Metabolic Status							
**Total cholesterol (TC) (mmol/L)**	3.16 (0.9)	3.3 (1.0)	0.14 (-0.1, 0.3)	0.14 (-0.1, 0.3	3.20 (0.8)	0.04 (-0.2, 0.3)	0.03 (-0.2, 0.2)
**TC:HDL ratio**	3.78 (1.5)	3.87 (1.9)	0.09 (-0.3, 0.4)	0.07 (-0.3, 0.4)	3.82 (1.4)	0.04 (-0.3, 0.4)	0.03 (-0.4, 0.4)
**Baseline glucose (n=50)**	4.60 (0.6)	4.90 (0.8)	0.30 (-0.1, 0.7)	0.50 * (0.0, 1.0)	4.71 (0.4)	0.11 (-0.5, 0.7)	0.14 (-0.6, 0.9)
**120 minutes glucose (n=49)**	5.80 (1.4)	5.99 (0.9)	0.19 (-0.5, 0.9)	-0.12 (-1.0, 0.7)	6.21 (1.5)	0.41 (-0.8, 1.6)	0.21 (-1.2, 1.6)
**Salivary cortisol (nmol/l) (n=62)**	4.22 (1.7)	4.82 (1.9)	0.61 (-0.4, 1.6)	0.57 (-0.5, 1.7)	5.08 (1.7)	0.87 (-0.3, 2.0)	0.86 (-0.4, 2.1)
**HbA1c (%)**	5.09 (0.5)	5.18 (0.5)	0.09 (-0.0, 0.2)	0.08 (-0.0, 0.2)	5.08 (0.5)	-0.01 (-0.1, 0.1)	-0.01 (-0.1, 0.1)

Adjusted difference includes age, sex and SES in regression model. Adjusted difference for lung function outcomes includes sitting height and puberty only

Web Annex 4: Results of unadjusted and adjusted regression analysis for growth and body composition outcomes (extension of Table 2)

Cases (n=378)		Sibling Controls (n=219)					Community Controls (n=184)				
	**Mean** (SD)	**Mean** (SD)	**Unadjusted difference** (95% CI)	***P value***	**Adjusted difference** (95% CI)	***P value***	**Mean** (SD)	**Unadjusted difference** (95% CI)	***P value***	**Adjusted difference** (95% CI)	***P value***
Growth indices											
**WAZ**	-1.6 (0.9)	-1.4 (1.0)	0.1 (-0.1, 0.4)	*0.27*	-0.2 (-0.5, 0.1)	*0.16*	-1.2 (0.9)	0.4 *(0.0, 0.5)	*<0.01*	0.3 (-0.0, 0.5)	*0.06*
**BAZ**	-0.8 (0.9)	-0.8 (0.9)	0.03 (-0.1, 0.2)	*0.71*	0.08 (-0.1, 0.3)	*0.39*	-0.7 (0.9)	0.1(-0.0, 0.3)	*0.14*	0.1 (-0.1, 0.3)	*0.31*
**HAZ**	-1.8 (1.2)	-1.5 (1.2)	0.3 * (0.1, 0.5)	*0.01*	0.2 *(0.0, 0.4)	*0.04*	-1.3 (1.1)	0.5 *(0.3, 0.7)	*<0.01*	0.4 *(0.2, 0.6)	*<0.01*
Height											
**Standing (cm)**	124.9 (9.0)	130.3 (16.8)	5.3 *(3.1, 7.5)	*<0.01*	2.0 *(0.6- 3.4)	*<0.01*	127.4 (9.9)	2.4 *(0.1, 4.8)	*0.04*	2.7 *(1.2, 4.2)	*<0.01*
**Sitting (cm)**	65.2 (4.4)	67.4 (7.6)	2.2 * (1.2,3.2)	*<0.01*	0.6 (-0.1,1.3)	*0.07*	65.9 (4.5)	0.8 (-0.3,1.9)	*0.18*	0.7 (-0.1,1.5)	*0.08*
**Leg length (cm)**	59.9 (5.5)	63.0 (9.6)	3.1 * (1.8, 4.4)	*<0.01*	1.4 * (0.5, 2.3)	*<0.01*	61.6 (6.0)	1.75*(0.3, 3.2)	*0.02*	2.0 *(1.0, 3.0)	*<0.01*
Circumferences											
**Head (cm)**	51.1 (2.1)	52.1 (2.5)	0.3 * (0.0, 0.7)	*0.04*	0.1 (-0.3, 0.5)	*0.31*	52.1 (1.9)	0.3(-0.1, 0.7)	*0.21*	0.3 (-0.1, 0.8)	*0.12*
**MUAC (mm)**	172 (20)	183 (29.8)	10.9 *(6.7, 15.1)	*<0.01*	5.7 *(2.3, 9.1)	*<0.01*	178 (22)	5.5*(1.0, 10.1)	*0.02*	5.6 *(1.9, 9.4)	*<0.01*
**Calf (cm)**	23.7 (2.3)	25.0 (3.5)	1.36 *(0.9, 1.9)	*<0.01*	0.62 *(0.2, 1.0)	*<0.01*	24.3 (2.4)	0.59*(0.2, 1.0)	*0.02*	0.49 *(0.1,0.9)	*0.01*
Body composition											
**R/h**	603.5 (105.1)	567.4 (130)	-36.1*(-56,-16.1)	*<0.01*	-11.5 (-29,-5.9)	*0.19*	577.1 (90.7)	-26.4*(-48, -4.6)	*0.02*	-24.5*(-43,-5.5)	*0.01*
**Xc/h**	52.2 (8.4)	49.7 (9.3)	-2.5 * (-4.1,-0.9)	*<0.01*	-0.5 (-2.1,1.0)	*0.52*	51.5 (7.8)	-0.7 (-2.3, 1.0)	*0.43*	0.3 (-1.4, 1.9)	*0.75*
**Phase angle ^{0}**	5.0 (0.7)	5.1 (0.7)	0.1 (-0.1,0.2)	*0.10*	0.1 (-0.0,0.2)	*0.16*	5.1 (0.5)	0.1 (-0.0,0.2)	*0.07*	0.2 *(0.1, 0.3)	*<0.01*
**Waist circumference**	56.3 (4.2)	57.7 (6.4)	1.45 * (0.5, 2.3)	*<0.01*	0.55 (-0.2, 1.3)	*0.15*	56.0 (4.5)	-0.25 (-1.2, 0.7)	*0.61*	0.08 (-0.7, 0.9)	*0.86*

**(cm)**											
**Hip circumference (cm)**	62.3 (5.8)	65.9 (10.2)	3.51 *(2.1, 4.9)	*<0.01*	1.83 *(0.8, 2.8)	*<0.01*	63.7 (6.9)	1.34 (-0.2, 2.8)	*0.08*	1.56 *(0.5, 2.7)	*0.01*
**Waist:hip ratio**	0.91 (0.1)	0.88 (0.1)	-0.02 *(-0.0,-0.0)	*<0.01*	-0.01 (-0.0,0.0)	*0.07*	0.89 (0.1)	-0.02 *(-0.0, -0.0)	*0.01*	-0.02 *(-0.0, -0.0)	*0.01*

Web Annex 5: Results of unadjusted and adjusted regression analysis for NCD risk factor outcomes (extension of Table 3)

Outcomes	Cases n=378	Sibling Controls n=219					Community Controls n=184				
	**Mean (SD)**	**Mean (SD)**	**Unadjusted difference (95% CI)**	***P value***	**Adjusted difference (95% CI)**	***P value***	**Mean (SD)**	**Unadjusted difference (95% CI)** ***p***	***P value***	**Adjusted difference (95% CI)**	***P value***
Lung Function											
**FEV_1 Z score**	-0.47 (1.1)	-0.48 (1.0)	-0.02 (-0.2, 0.2)	*0.88*	-0.02 (-0.3, 0.2)	*0.88*	-0.34 (1.1)	0.13(-0.1, 0.4)	*0.30*	0.10 (-0.2, 0.4)	*0.46*
**FVC Z score**	-0.32 (1.0)	-0.38 (1.1)	-0.06 (-0.3, 0.2)	*0.61*	-0.11(-0.4, 0.1)	*0.41*	-0.15 (1.1)	0.17 (-0.1, 0.4)	*0.18*	0.12 (-0.1, 0.1)	*0.36*
**FEV_1/FVC ratio**	-0.21 (0.9)	-0.15 (0.9)	0.06 (-0.1, 0.3)	*0.54*	0.14 (-0.1, 0.4)	*0.25*	-0.37 (1.0)	-0.16 (-0.4, 0.1)	*0.14*	-0.13 (-0.4, 0.1)	*0.32*
Physical function											
**Hand grip strength (kg)**	12.7 (6.3)	14.8 (7.9)	2.05 * (1.2, 2.9)	*<0.01*	1.01 * (0.3, 1.7)	*0.01*	13.8 (3.9)	1.39 *(0.4, 2.4)	*0.01*	1.68 * (0.9, 2.4)	*<0.01*
**Steps per hour (n=78)**	716.5 (413)	955.8 (361)	141.5 (-10,293)	*0.07*	144.5 (-37,325)	*0.12*	770.3 (367)	24.6 (-116,166)	*0.73*	-6.72(-185,172)	*0.94*
Cardiovascular Health											
**Total cholesterol(TC) (mmol/L)**	3.23 (0.9)	3.29 (1.0)	0.05 (-0.1, 0.2)	*0.55*	0.02 (-0.2,0.2)	*0.81*	3.19 (0.8)	-0.04 (-0.2, 0.2)	*0.71*	-0.08(-0.3, 0.1)	*0.46*
**TC:HDL ratio**	3.69 (1.5)	3.87 (1.9)	0.17 (-0.1, 0.5)	*0.27*	-0.04 (-0.4,0.3)	*0.81*	3.82 (1.4)	0.12 (-0.2, 0.5)	*0.49*	-0.13 (-0.5, 0.3)	*0.51*
**BP systolic (mmHg)**	107.8 (9.5)	109.5 (11.4)	1.69 (-0.1, 3.5)	*0.07*	-0.17 (-2.0,1.6)	*0.86*	108.3 (9.4)	0.45 (-1.5, 2.4)	*0.66*	0.17 (-1.8, 2.2)	*0.87*
**BP diastolic (mmHg)**	69.9 (8.6)	68.8 (9.6)	-1.18 (-2.8, 0.4)	*0.15*	-1.91 *(-3.6,-0.2)	*0.03*	68.4 (9.0)	-1.59 (-3.3, 0.2)	*0.08*	-1.60 (-3.5, 0.3)	*0.10*
Metabolic											
**Baseline glucose (n=59)**	4.54 (0.6)	4.81 (0.8)	0.27 (-0.1, 0.6)	*0.15*	0.59 *(0.1, 1.1)	*0.02*	4.71 (0.4)	0.17 (-0.4,0.8)	*0.58*	0.25(-0.5, 1.0)	*0.52*
**120 minutes glucose (n=56)**	5.54 (1.3)	5.93 (0.9)	0.39 (-0.3, 1.1)	*0.26*	-0.20 (-1.1, 0.7)	*0.65*	6.21 (1.5)	0.67 (-0.5, 1.8)	*0.26*	0.13(-1.3, 1.6)	*0.86*

**Salivary Cortisol (nmol/l) (n=82)**	4.49 (1.7)	4.73 (1.8)	0.23 (-0.6, 1.1)	*0.59*	0.32 (-0.7,1.3)	*0.53*	5.08 (1.7)	0.59 (-0.5, 1.7)	*0.28*	0.77 (-0.6,2.1)	*0.26*
**HbA1c (%)**	5.13 (0.5)	5.17 (0.5)	0.04 (-0.1, 0.1)	*0.46*	0.05 (-0.1,0.2)	*0.40*	5.07 (0.5)	-0.07 (-0.2, 0.0)	*0.25*	-0.06 (-0.2, 0.1)	*0.36*

Web Annex 6: Expanded Methods

Verbal descriptions of the geographic locations of their homes collected in 2006 were used to locate case children, after which control children were recruited. Basic demographic information and anthropometric data (listed below) were collected at participants' homes. Further outcomes were then collected at a hospital appointment. The data recording took place on mobile phones (Samsung Galaxy S) using an ODK-based open-source platform (www.commcarehq.org). This allowed on-the-spot calculations of anthropometry quality control indicators and questionnaires were coded to ensure that the correct skip-logic was applied, with no relevant questions left blank.

Our main outcome variables were: anthropometry; body composition; lung function; physical capacity (hand grip; step test; physical activity); and blood markers of NCD risk. Exposures included: previous malnutrition; socioeconomic and family status; HIV; and parental education.

Anthropometric assessments followed guidelines by Lohman et al., and WHO[1, 2] and were subject to quality control which involved two members of the trained study team taking independent readings. Both observers repeated their measurements if they were not within the limits of agreement, as used in the WHO Multicentre Growth Reference Study[2]. Body weight was measured only once using Marsden digital medical scales to the nearest 0.1kg (MS4202L, Marsden Weighing Group, UK). Height, sitting height, and lower limb length were measured using a Leicester Height Measure (HM-250P, Marsden Weighing Group, UK) to the nearest 0.1cm; a specially designed "sitting height stool" was used which accommodated the base of the height measure for the child to sit on and had adjustable foot rests.

Following anthropometry, participants were asked to attend a hospital appointment a few days later for measurement of body composition, muscle strength, physical capacity, glucose tolerance, lipid profile, salivary cortisol and blood pressure. Body composition was measured using skinfold thickness measured at biceps, triceps, subscapular, and suprailiac sites on the left side with Tanner/Whitehouse callipers (Holtain Ltd, UK), and using bioelectrical impedance analysis (BIA)

(Quadscan 4000; Bodystat Ltd, UK). Both skinfold thickness and BIA readings were measured in duplicate. The Quadscan provides tetrapolar data on impedance, resistance, reactance and phase angle using measurement at 50 kHz [3]. Results are presented as Resistance index (R/height) and Reactance index (Xc/height). Usually BIA outputs are converted to total body water and fat free mass using population-specific empirical equations [4-6]. In the absence of a population-specific equation, it is possible to assess relative hydration and lean mass using raw BIA values adjusted for height.

Physical activity was measured on a subset of children using Actilife accelerometers worn for at least 48 consecutive hours over 3-5 days (Actigraph corp, USA)[7]. Although the accelerometers can provide complex information such as proportion of the day spent in sedentary/active state, these outputs use cut-off values which are not validated in this population; hence we simply used "steps per day" as the physical activity outcome. Muscle strength was measured using "Takei Grip-D" (Takei Scientific Instruments Co Ltd, Japan) device which measures hand grip strength from 5kg upwards. The best of three attempts on either hand was used in final analysis. Physical capacity was measured using the iStep test (incremental step test) developed by the UCL Institute of Child Health[8] where the participant is required to step up and down to a beep of increasing speed for up to 10 minutes whilst measuring their heart rate and oxygen saturation. Lung function was measured using spirometry on an Easy-On PC device (NDD Medical, Switzerland); quality grades were applied to the data for each child based on repeatability of technically accepted traces; quality control was undertaken by a blinded, senior respiratory physiologist at UCL Institute for Child Health (Respiratory Unit)[9]. The three main spirometry outcomes were: forced expiratory volume in 1 second (FEV_1), forced vital capacity (FVC) and the FEV_1/FVC ratio expressed as z-scores, based on the Global Lung function Initiative (GLI) Black reference equations (based on the African-American population), which adjusts for height, age, sex and ethnicity[10].

Lipid profile and full blood count were measured using venous blood taken in the morning after 12 hours of fasting. Serum for analysis of lipid profile was frozen at -80◦C and thawed after 30 days for analysis using an Enzymatic Colorimetric method (Beckman Coulter analyser Model-AU480). Percentage HbA1c was measured using a Beckman Coulter AU analyzer; HbA1c levels are an

indication of average blood sugar levels and therefore an indicator of diabetes risk. Salivary cortisol was assessed mid-morning using SalivaBio Oral swabs and protocol (Salimetrics, USA)[11]. Blood pressure was measured using an OMRON model BP710N with a child-sized arm cuff (OMRON Healthcare Europe B.V., The Netherlands). A subset of children (n=60) also underwent an oral glucose tolerance test using 1.75mg per kg body weight of Polycal glucose powder (Nutricia Ltd, Ireland); venous glucose was measured at baseline, 30, 60 and 120 minutes post-ingestion[12]. Standard WHO cut-offs for fasting glycaemia and impaired glucose tolerance were applied[13].

Data collectors were not blinded to the case/control status of the children due to study logistics. HIV status was established from official results in health passports (for both participant and mother); if their status was not known, a HIV test was offered during the hospital appointment by a trained counsellor. For those who did not consent to a test, HIV status was recorded as unknown. Puberty was simply recorded as a binary variable, as reported by the participant or guardian (onset of menarche in girls, voice change in boys).

Socio-economic status (SES) was derived from asset scores using Malawi Demographic Health Survey (DHS) questions[14]. DHS questions were also used to assess water and sanitation facilities, location of cooking facilities, and normal cooking fuel used.

Web Annex 7: Results of unadjusted BIA Vector analysis (BIVA)

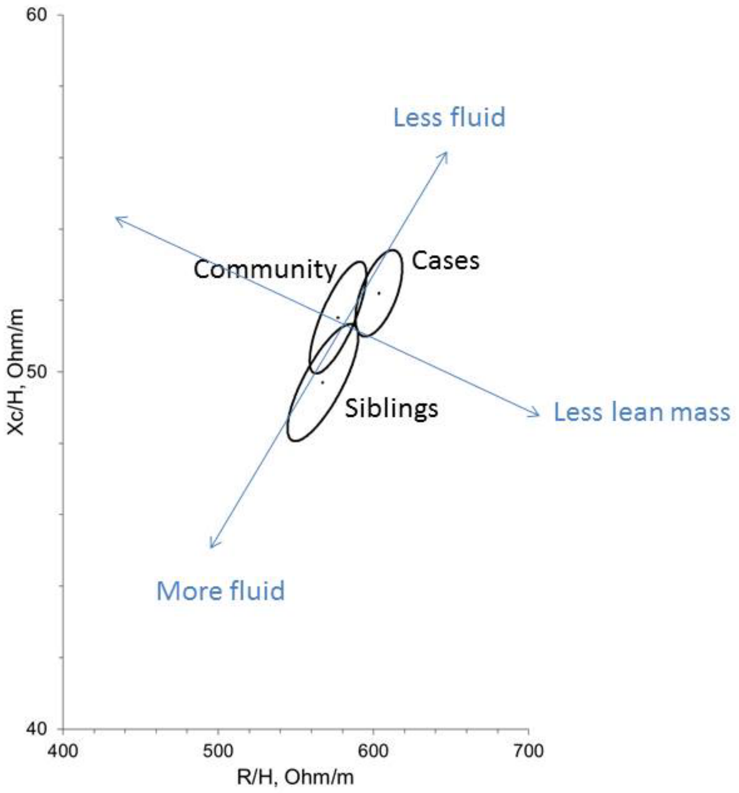

*Xc is reactance; R is resistance. Both Xc and R are divided by height (H). Cases have more lean mass and more fluid that community controls; although siblings also have significantly different body composition to cases, these differences are all explained by age difference between cases and siblings.

References

1. Lohman T, Roche A, Martorell R. Anthropometric standardization reference manual. Human Kinetics Books. Champaign, IL1988.
2. de Onis M, Garza C, Victora CG, Onyango AW, Frongillo EA, Martines J. The WHO Multicentre Growth Reference Study: planning, study design, and methodology. Food & Nutrition Bulletin. 2004;25(Supplement 1):15S-26S.
3. Girma T, Kæstel P, Workeneh N, Mølgaard C, Eaton S, Andersen GS, et al. Bioimpedance index for measurement of total body water in severely malnourished children: Assessing the effect of nutritional oedema. Clinical Nutrition. 2015.
4. Kotler DP, Burastero S, Wang J, Pierson R. Prediction of body cell mass, fat-free mass, and total body water with bioelectrical impedance analysis: effects of race, sex, and disease. The American journal of clinical nutrition. 1996;64(3):489S-97S.
5. Jackson A, Pollock ML, Graves JE, Mahar MT. Reliability and validity of bioelectrical impedance in determining body composition. Journal of Applied Physiology. 1988;64(2):529-34.
6. Lukaski HC, Johnson PE, Bolonchuk WW, Lykken GI. Assessment of fat-free mass using bioelectrical impedance measurements of the human body. The American journal of clinical nutrition. 1985;41(4):810-7.
7. Pulakka A, Cheung Y, Ashorn U, Penpraze V, Maleta K, Phuka J, et al. Feasibility and validity of the ActiGraph GT3X accelerometer in measuring physical activity of Malawian toddlers. Acta paediatrica. 2013;102(12):1192-8.
8. Rand S, Prasad A, Main E. New incremental field step-test (i-step) is valid and feasible in measuring near maximal exercise performance in children with cystic fibrosis. Physiotherapy Elsevier 2015.
9. Kirkby J, Welsh L, Lum S, Fawke J, Rowell V, Thomas S, et al. The EPICure study: comparison of pediatric spirometry in community and laboratory settings. Pediatric pulmonology. 2008;43(12):1233-41.
10. Quanjer PH, Stanojevic S, Cole TJ, Baur X, Hall GL, Culver BH, et al. Multi-ethnic reference values for spirometry for the 3–95-yr age range: the global lung function 2012 equations. European Respiratory Journal. 2012;40(6):1324-43.
11. Salimetrics L, Europe S. Saliva collection and handling advice. Available oat www salimetrics com Accessed. 2011;1.
12. Wabitsch M, Hauner H, Hertrampf M, Muche R, Hay B, Mayer H, et al. Type II diabetes mellitus and impaired glucose regulation in Caucasian children and adolescents with obesity living in Germany. Int J Obes Relat Metab Disord. 2004;28(2):307-13.
13. Alberti KGMM, Zimmet Pf. Definition, diagnosis and classification of diabetes mellitus and its complications. Part 1: diagnosis and classification of diabetes mellitus. Provisional report of a WHO consultation. Diabetic medicine. 1998;15(7):539-53.
14. National Statistical Office (NSO), Macro I. Malawi Demographic and Health Survey 2010. Zomba Malawi, and Maryland, USA: 2011.
